# Editorial: Cancer and inflammatory diseases research: from the basics to the precision medicine

**DOI:** 10.3389/fphar.2024.1477963

**Published:** 2024-08-27

**Authors:** Adriano Sabino, Masamitsu Kanada, Elaine Leite, Mariana Quezado, Whocely Victor de Castro

**Affiliations:** ^1^ Department of Clinical and Toxicological Analysis, Faculty of Pharmacy, Federal University of Minas Gerais, Belo Horizonte, Brazil; ^2^ Institute for Quantitative Health Science and Engineering (IQ), Michigan State University, East Lansing, MI, United States; ^3^ Department of Pharmacology and Toxicology, Michigan State University, East Lansing, MI, United States; ^4^ College of Human Medicine, Michigan State University, East Lansing, MI, United States; ^5^ Department of Pharmaceutical Products, Faculty of Pharmacy, Federal University of Minas Gerais, Belo Horizonte, Brazil; ^6^ Department of Biochemistry and Immunology, Institute of Biological Sciences, Federal University of Minas Gerais, Belo Horizonte, Brazil; ^7^ Pharmaceutical Sciences Program, Federal University of São João del-Rei, São João del Rei, Brazil

**Keywords:** cancer, inflamatory diseases, therapy, precision medicine, pharmacology

## 1 Introduction

The field of pharmacology is rapidly evolving, with significant advancements in our understanding of cancer and inflammatory diseases. This editorial highlights nine groundbreaking studies exemplifying the transition from basic research to precision medicine, offering novel insights and potential therapeutic strategies for these challenging conditions.

### 1.1 Exosomes and colorectal cancer

Exosomes, extracellular vesicles rich in bioactive substances such as DNA, RNA, lipids, and proteins, play a crucial role in cell-to-cell communication and the pathogenesis of colorectal cancer (CRC). A comprehensive review focuses on the relatively unexplored area of exosomal genomic DNA (gDNA) and its significance in CRC. Exosome gDNA, which includes clinically relevant tumor-specific mutation genes, is pivotal in liquid biopsy applications for early diagnosis and treatment. Additionally, exosome gDNA influences immune and metabolic functions in CRC, positioning it as a critical target for future research and clinical interventions (Li et al.).

### 1.2 PARP inhibitors in cutaneous squamous cell carcinoma

Poly ADP-ribose polymerase inhibitors (PARPis) have been effective in treating BRCA1/2 mutation-related cancers. A case study of a 40-year-old man with metastatic cutaneous squamous cell carcinoma (cSCC) with a BRCA2 mutation demonstrated the efficacy of PARPi fluzoparib. This treatment resulted in tumor stability and progression-free survival of 5 months, suggesting that PARPis can be a viable option for cSCC patients with BRCA mutations, thereby expanding the therapeutic options for this patient population (Sun et al.).

### 1.3 Mortality analysis in breast cancer patients

An extensive study on breast cancer patients highlights the importance of understanding mortality from various causes to improve healthcare planning and clinical predictions. The research, involving 12,742 women, revealed that breast cancer is the leading cause of death, followed by cardiovascular disease. Notably, the contribution of breast cancer to overall mortality varied significantly by age and disease stage, emphasizing the need for age-specific and stage-specific survivorship care models that incorporate multidisciplinary approaches (Contiero et al.).

### 1.4 Foam-based intraperitoneal chemotherapy

Innovative approaches in drug delivery, such as foam-based intraperitoneal chemotherapy (FBIC), are being explored to improve treatment efficacy and safety. A study on swine models demonstrated the feasibility and safety of using doxorubicin in FBIC. The results showed promising intraoperative and postoperative outcomes without significant complications, suggesting that FBIC could be a viable alternative to traditional liquid solutions for intraperitoneal chemotherapy, pending further long-term studies (Khosrawipour et al.).

### 1.5 Monitoring cellular immunotherapy with synthetic notch receptors

Cellular immunotherapy, particularly CAR T cell therapy, has revolutionized cancer treatment. However, monitoring these therapies remains challenging. Researchers have developed a synthetic Notch (synNotch) receptor system that links immune-cancer cell interactions to a simple blood test. This system allows for the detection of intratumoral activity via a secreted reporter, providing a convenient and effective method to monitor the efficacy of immunotherapies and potentially improving patient-specific treatment strategies (Fu et al.).

### 1.6 Janus kinase inhibitors: from autoimmune diseases to cancer therapy

Janus kinase (JAK) inhibitors, initially developed for autoimmune diseases, have shown promise in cancer therapy due to their role in cytokine signaling pathways involved in tumorigenesis. A systematic review of JAK inhibitors highlighted their anti-tumor potential and clinical applicability. The review underscores the importance of further studies to optimize their use in oncology, potentially offering new therapeutic avenues for cancer patients (Wei and Liu).

### 1.7 Therapeutic drug monitoring of adalimumab

Adalimumab, a widely used biologic for inflammatory diseases, benefits from therapeutic drug monitoring (TDM) to optimize treatment outcomes. A systematic review assessed the impact of TDM on adalimumab therapy, finding that proactive TDM can guide dose adjustments and improve clinical responses. However, the evidence was not statistically significant, highlighting the need for more robust studies to validate the role of TDM in managing adalimumab therapy effectively (Li et al.).

### 1.8 Ferroptosis and the ubiquitin-proteasome system in cancer

Ferroptosis, an iron-dependent form of cell death, has emerged as a potential target for cancer therapy. Recent research explores the interplay between ferroptosis and the ubiquitin-proteasome system (UPS), which regulates protein stability. The study highlights key regulators of ferroptosis, such as GPX4 and NRF2, and their modulation by the UPS. Understanding this relationship could lead to novel therapeutic strategies that harness ferroptosis to combat cancer (Din et al.).

### 1.9 Clobenpropit and the CXCL12/CXCR4 axis

Clobenpropit, a histamine H3 receptor antagonist, has shown potential in treating autoimmune diseases and cancer by inhibiting the CXCR4 receptor. The CXCL12/CXCR4 axis is crucial in various biological processes, including cell proliferation, migration, and inflammation. A review of Clobenpropit’s effects on this pathway suggests that it could be an effective therapeutic agent for managing diseases like juvenile idiopathic arthritis and certain cancers, providing a new avenue for targeted therapy (Abbasifard et al.).

## 2 Conclusion

The articles featured in this Research Topic highlight the diverse and innovative approaches being explored in cancer and inflammatory disease research. From the molecular mechanisms of exosomes and ferroptosis to the clinical applications of PARPis and JAK inhibitors, these studies underscore the importance of integrating basic research with clinical practice. As precision medicine continues to evolve, these advancements will play a crucial role in developing more effective and personalized treatments, ultimately improving patient outcomes and transforming healthcare.

